# Fixed or mixed: a comparison of three, four and mixed-option multiple-choice tests in a Fetal Surveillance Education Program

**DOI:** 10.1186/1472-6920-13-35

**Published:** 2013-03-04

**Authors:** Nathan Zoanetti, Mark Beaves, Patrick Griffin, Euan M Wallace

**Affiliations:** 1 Assessment Research Centre, Melbourne Graduate School of Education, University of Melbourne, Parkville, Australia; 2 Royal Australian and New Zealand College of Obstetricians and Gynaecologists, East Melbourne, Australia; 3 Department of Obstetrics and Gynaecology, The Ritchie Centre, Monash Institute of Medical Research and Southern Clinical School, Monash University, Clayton, Australia

## Abstract

**Background:**

Despite the widespread use of multiple-choice assessments in medical education assessment, current practice and published advice concerning the number of response options remains equivocal. This article describes an empirical study contrasting the quality of three 60 item multiple-choice test forms within the Royal Australian and New Zealand College of Obstetricians and Gynaecologists (RANZCOG) Fetal Surveillance Education Program (FSEP). The three forms are described below.

**Methods:**

The first form featured four response options per item. The second form featured three response options, having removed the least functioning option from each item in the four-option counterpart. The third test form was constructed by retaining the best performing version of each item from the first two test forms. It contained both three and four option items.

**Results:**

Psychometric and educational factors were taken into account in formulating an approach to test construction for the FSEP. The four-option test performed better than the three-option test overall, but some items were improved by the removal of options. The mixed-option test demonstrated better measurement properties than the fixed-option tests, and has become the preferred test format in the FSEP program. The criteria used were reliability, errors of measurement and fit to the item response model.

**Conclusions:**

The position taken is that decisions about the number of response options be made at the item level, with plausible options being added to complete each item on both psychometric and educational grounds rather than complying with a uniform policy. The point is to construct the better performing item in providing the best psychometric and educational information.

## Background

Several studies provide advice on the matter of the optimal number of multiple-choice question response options [1-4]. There are also several medical education assessment programs with long-standing policies and practices of their own [5,6]. What becomes apparent is that recommendations are generally conditional upon assumptions and contextual factors in each assessment setting. What follows is a review of a number of prominent approaches regarding the number of MCQ response options.

**Table 1:** Item statistics for the four-option and the three-option test forms (removed distractors are highlighted)

	**Four-option form**											**Three-option form**								
**Item**	**Facility**	**r**	**A %**	**A Pt Bis**	**B %**	**B Pt Bis**	**C %**	**C Pt Bis**	**D %**	**D Pt Bis**	**% Miss**	**Facility**	**r**	**A %**	**A Pt Bis**	**B %**	**B Pt Bis**	**C %**	**C Pt Bis**	**% Miss**
1	9.96	0.07	20.58	-0.10	**4.98**	**-0.01**	64.35	0.04	9.96	0.07	0.13	14.55	0.13	11.61	-0.16	73.81	0.01	14.55	0.13	0.00
2	68.55	0.36	28.57	-0.32	2.10	-0.13	**0.26**	**-0.01**	68.55	0.36	0.52	78.95	0.27	19.66	-0.27	1.39	-0.03	78.95	0.27	0.00
3	71.17	0.33	11.80	-0.05	71.17	0.33	10.48	-0.20	**5.90**	**-0.27**	0.66	76.47	0.29	15.17	-0.10	76.47	0.29	8.20	-0.30	0.15
4	65.53	0.30	65.53	0.30	5.64	-0.14	**2.62**	**-0.12**	25.16	-0.18	1.05	68.27	0.36	68.27	0.36	9.91	-0.24	20.28	-0.21	1.55
5	65.27	0.27	9.31	-0.21	65.27	0.27	**8.26**	**-0.11**	16.51	-0.08	0.66	78.79	0.33	11.15	-0.26	78.79	0.33	9.91	-0.16	0.15
6	60.81	0.35	19.13	-0.31	17.43	-0.09	60.81	0.35	**2.10**	**-0.05**	0.52	72.91	0.26	12.07	-0.21	72.91	0.26	14.86	-0.12	0.15
7	87.02	0.33	4.59	-0.18	**1.57**	**-0.13**	87.02	0.33	6.42	-0.21	0.39	84.21	0.34	6.19	-0.21	84.21	0.34	9.29	-0.24	0.31
8	60.55	0.37	**1.83**	**-0.13**	31.19	-0.26	6.42	-0.19	60.55	0.37	0.00	62.73	0.28	34.01	-0.22	2.95	-0.18	62.73	0.28	0.31
9	3.28	0.01	53.21	-0.20	3.28	0.01	43.25	0.20	**0.13**	**-0.01**	0.13	4.64	0.05	48.76	-0.14	4.64	0.05	46.13	0.13	0.46
10	77.06	0.43	4.85	-0.16	**3.28**	**-0.15**	14.81	-0.34	77.06	0.43	0.00	81.89	0.31	4.95	-0.22	12.85	-0.20	81.89	0.31	0.31
11	73.92	0.37	14.94	-0.22	**2.10**	**-0.07**	73.92	0.37	8.91	-0.25	0.13	80.80	0.39	12.54	-0.25	80.80	0.39	6.66	-0.28	0.00
12	82.96	0.19	13.63	-0.12	**1.18**	**-0.08**	1.57	-0.13	82.96	0.19	0.66	87.93	0.19	9.44	-0.12	2.48	-0.19	87.93	0.19	0.15
13	67.10	0.42	16.38	-0.34	**2.75**	**-0.10**	67.10	0.42	13.76	-0.16	0.00	76.16	0.41	13.47	-0.38	76.16	0.41	10.06	-0.12	0.31
14	65.40	0.41	**0.79**	**-0.05**	65.40	0.41	8.26	-0.32	25.29	-0.24	0.26	80.03	0.33	80.03	0.33	4.95	-0.30	14.71	-0.20	0.15
15	57.54	0.17	19.79	-0.18	**4.46**	**0.03**	17.96	-0.05	57.54	0.17	0.26	60.06	0.22	21.36	-0.17	17.96	-0.09	60.06	0.22	0.62
16	76.80	0.23	**7.08**	**-0.10**	8.26	-0.10	76.80	0.23	7.86	-0.16	0.00	83.44	0.28	5.88	-0.14	83.44	0.28	10.68	-0.23	0.00
17	27.39	0.20	37.75	-0.21	34.21	0.04	27.39	0.20	**0.66**	**-0.06**	0.00	28.48	0.27	31.58	-0.22	39.63	-0.05	28.48	0.27	0.31
18	60.94	0.30	10.88	-0.19	**13.11**	**-0.21**	60.94	0.30	15.07	-0.04	0.00	63.47	0.31	19.66	-0.25	63.47	0.31	16.72	-0.13	0.15
19	89.38	0.25	**2.88**	**-0.20**	4.19	-0.07	89.38	0.25	3.41	-0.15	0.13	93.03	0.20	4.18	-0.08	93.03	0.20	2.79	-0.21	0.00
20	83.49	0.35	5.11	-0.23	83.49	0.35	7.21	-0.21	**3.80**	**-0.09**	0.39	90.71	0.31	90.71	0.31	5.11	-0.26	4.02	-0.17	0.15
21	44.82	0.38	41.94	-0.26	9.04	-0.09	**4.06**	**-0.16**	44.82	0.38	0.13	52.94	0.31	39.32	-0.19	7.59	-0.23	52.94	0.31	0.15
22	62.91	0.33	27.00	-0.25	62.91	0.33	6.03	-0.10	**3.93**	**-0.11**	0.13	60.37	0.30	31.42	-0.18	60.37	0.30	7.74	-0.21	0.46
23	80.60	0.33	5.64	-0.21	**1.70**	**-0.09**	11.93	-0.21	80.60	0.33	0.13	87.46	0.37	1.24	-0.11	10.99	-0.33	87.46	0.37	0.31
24	40.63	0.36	37.48	-0.11	40.63	0.36	15.73	-0.23	**5.90**	**-0.17**	0.26	46.59	0.34	39.78	-0.17	46.59	0.34	13.47	-0.25	0.15
25	91.61	0.19	**1.05**	**-0.06**	1.57	-0.06	91.61	0.19	5.64	-0.16	0.13	93.19	0.27	1.86	-0.18	93.19	0.27	4.64	-0.18	0.31
26	85.98	0.36	3.15	-0.24	10.48	-0.26	85.98	0.36	**0.39**	**-0.05**	0.00	82.97	0.36	3.72	-0.15	13.16	-0.32	82.97	0.36	0.15
27	72.35	0.25	72.35	0.25	**9.04**	**-0.23**	12.06	-0.06	5.90	-0.07	0.66	77.24	0.18	77.24	0.18	12.54	-0.16	9.91	-0.06	0.31
28	49.54	0.46	**6.82**	**-0.16**	37.61	-0.25	49.54	0.46	5.77	-0.28	0.26	57.28	0.44	37.62	-0.32	57.28	0.44	4.95	-0.30	0.15
29	49.93	0.18	2.75	-0.14	**1.31**	**-0.08**	49.93	0.18	45.87	-0.12	0.13	54.18	0.17	2.32	-0.12	54.18	0.17	43.50	-0.13	0.00
30	42.86	0.26	41.68	-0.04	11.40	-0.23	42.86	0.26	**3.80**	**-0.18**	0.26	41.95	0.24	53.72	-0.17	41.95	0.24	4.18	-0.16	0.15
31	72.74	0.33	8.39	-0.22	72.74	0.33	**10.22**	**-0.16**	8.39	-0.13	0.26	85.29	0.27	6.50	-0.21	85.29	0.27	8.20	-0.15	0.00
32	63.17	0.17	63.17	0.17	28.83	-0.09	3.54	-0.21	**4.46**	**-0.01**	0.00	70.74	0.27	70.74	0.27	25.85	-0.22	3.10	-0.13	0.31
33	49.67	0.28	9.17	-0.11	**28.57**	**-0.16**	12.19	-0.09	49.67	0.28	0.39	61.92	0.16	16.10	-0.13	21.67	-0.07	61.92	0.16	0.31
34	39.71	0.27	31.98	-0.14	39.71	0.27	**9.31**	**-0.17**	18.22	-0.04	0.79	59.60	0.36	25.08	-0.29	59.60	0.36	15.33	-0.14	0.00
35	77.72	0.27	1.44	-0.14	77.72	0.27	19.53	-0.21	**1.18**	**-0.13**	0.13	80.34	0.32	80.34	0.32	17.80	-0.27	1.70	-0.18	0.15
36	44.30	0.29	38.14	-0.14	**5.50**	**-0.06**	12.06	-0.19	44.30	0.29	0.00	54.64	0.31	35.14	-0.21	9.91	-0.19	54.64	0.31	0.15
37	25.43	0.21	25.43	0.21	**10.35**	**-0.03**	13.89	-0.05	49.54	-0.11	0.79	52.17	0.30	52.17	0.30	10.53	-0.14	37.31	-0.22	0.00
38	80.08	0.35	80.08	0.35	5.24	-0.13	**3.28**	**-0.13**	11.27	-0.27	0.13	80.50	0.31	80.50	0.31	5.88	-0.17	13.47	-0.23	0.15
39	92.92	0.23	2.49	-0.16	**0.52**	**-0.09**	2.23	-0.15	92.92	0.23	1.83	96.28	0.16	0.77	-0.09	2.17	-0.08	96.28	0.16	0.77
40	69.07	0.29	5.90	-0.15	**1.97**	**-0.02**	69.07	0.29	22.94	-0.22	0.13	61.30	0.30	5.73	-0.17	61.30	0.30	32.97	-0.23	0.00
41	71.30	0.22	6.29	-0.09	71.30	0.22	**3.15**	**-0.09**	19.13	-0.15	0.13	86.69	0.24	8.36	-0.17	86.69	0.24	4.64	-0.12	0.31
42	92.66	0.21	1.97	-0.21	3.41	-0.07	92.66	0.21	**1.57**	**-0.09**	0.39	95.05	0.12	2.17	-0.16	2.63	0.00	95.05	0.12	0.15
43	77.20	0.25	4.06	-0.13	16.25	-0.14	77.20	0.25	**2.49**	**-0.18**	0.00	85.29	0.21	2.94	-0.16	11.76	-0.14	85.29	0.21	0.00
44	46.26	0.44	42.07	-0.30	46.26	0.44	7.99	-0.24	**3.67**	**-0.04**	0.00	48.92	0.40	43.96	-0.25	48.92	0.40	6.97	-0.30	0.15
45	52.95	0.20	52.95	0.20	21.49	-0.13	9.44	-0.16	**15.86**	**0.01**	0.26	62.54	0.36	62.54	0.36	28.02	-0.26	9.29	-0.18	0.15
46	53.87	0.20	**7.08**	**-0.02**	53.87	0.20	11.53	-0.20	27.26	-0.07	0.26	68.42	0.23	68.42	0.23	11.46	-0.27	19.66	-0.06	0.46
47	69.33	0.32	16.51	-0.22	69.33	0.32	**5.77**	**-0.19**	8.39	-0.07	0.00	72.76	0.40	14.09	-0.28	72.76	0.40	12.85	-0.23	0.31
48	55.44	0.45	20.05	-0.20	**6.95**	**-0.27**	55.44	0.45	17.30	-0.19	0.26	65.02	0.42	14.24	-0.25	65.02	0.42	20.59	-0.28	0.15
49	87.68	0.24	4.33	-0.14	87.68	0.24	4.85	-0.09	**2.88**	**-0.16**	0.26	85.76	0.30	5.57	-0.20	85.76	0.30	8.51	-0.21	0.15
50	47.84	0.18	**5.77**	**-0.16**	20.45	-0.07	47.84	0.18	25.29	-0.03	0.66	68.58	0.17	9.60	-0.19	68.58	0.17	21.67	-0.06	0.15
51	73.79	0.34	9.17	-0.17	**3.80**	**-0.11**	11.66	-0.20	73.79	0.34	1.57	84.98	0.28	6.66	-0.13	7.89	-0.25	84.98	0.28	0.46
52	77.06	0.27	5.77	-0.21	77.06	0.27	5.77	0.02	**10.88**	**-0.20**	0.52	87.93	0.22	6.66	-0.19	87.93	0.22	5.11	-0.11	0.31
53	44.17	0.32	**1.97**	**-0.16**	25.16	-0.22	44.17	0.32	28.18	-0.08	0.52	53.25	0.14	19.20	-0.10	53.25	0.14	27.24	-0.07	0.31
54	42.99	-0.07	42.99	-0.07	13.76	-0.02	41.81	0.11	**0.66**	**-0.02**	0.79	48.30	0.00	48.30	0.00	50.31	0.02	1.08	-0.03	0.31
55	77.98	0.19	**1.44**	**-0.11**	8.52	-0.19	77.98	0.19	11.27	0.00	0.79	72.45	0.08	5.73	-0.17	72.45	0.08	21.52	0.01	0.31
56	39.71	0.30	15.47	-0.23	37.88	-0.12	39.71	0.30	**5.90**	**0.02**	1.05	45.98	0.35	11.92	-0.24	41.49	-0.18	45.98	0.35	0.62
57	69.99	0.35	25.16	-0.28	69.99	0.35	2.75	-0.14	**1.18**	**-0.02**	0.92	74.46	0.35	23.68	-0.30	74.46	0.35	1.55	-0.14	0.31
58	47.71	0.13	3.67	-0.18	43.77	0.00	47.71	0.13	**3.93**	**-0.11**	0.92	32.97	0.06	4.95	-0.21	61.76	0.04	32.97	0.06	0.15
59	72.08	0.17	72.08	0.17	13.24	-0.18	10.48	0.04	**3.15**	**-0.08**	1.05	68.89	0.10	11.30	0.06	68.89	0.10	19.50	-0.16	0.31
60	93.71	0.16	2.36	-0.03	93.71	0.16	**0.79**	**-0.01**	2.10	-0.14	1.05	92.72	0.22	4.33	-0.15	92.72	0.22	2.63	-0.13	0.31

**Table 2:** Test statistics for the 48-item subsets from the three test forms

	**Three-option**	**Four-option**	**Mixed-option**
Classical reliability, α	0.78	0.79	0.82
Mean discrimination (SD)	0.30 (0.08)	0.30 (0.08)	0.32 (0.08)
Mean facility (SD)	70.8 (15.6)	63.8 (17.1)	71.4 (14.1)

The first approach to specifying multiple-choice option numbers might be termed *traditional*, where four or five options are used by convention [3]. Tarrant, Ware and Mohammed [3] explained that in many organisations the number of response options is uniformly fixed across all questions, and that this policy has little if any psychometric grounding. This is prominent in medical education assessments despite studies advocating the benefits of using fewer or varied option numbers [5,7]. Common four- and five-option approaches have certain drawbacks, especially where plausible alternatives become difficult to construct.

The next approach is to take the emergent majority recommendation, which might be termed the *meta-analytical* convention, based on empirical and theoretical studies. The consensus here is that three options is optimal [4,8,9]. This recommendation is often based on assumptions that the time taken to respond to three-option items is proportionally less than the time taken to respond to four-option items, as determined by the number of options alone [8]. Therefore some of the advocates of a three-option approach base their standpoint in part on the benefits of being able to construct and administer a larger number of items per unit time, thereby increasing content coverage and potentially increasing test reliability [9]. However, this assumption has been refuted on several occasions with the recognition that several other features of an item will influence response time [6,10,11]. Another argument in favour of a three-option policy relates to plausibility of options. Several studies have concluded that four- or five-option items rarely contain a full set of plausible alternatives [3]. Options attracting fewer than 5% of total respondents (or non positively discriminating distractors) are often classified as ‘non-functioning’ and this has often led to the recommendation for their removal [12,13].

When the number of items is fixed and the number of options is manipulated, mixed results and recommendations are reported; while some studies have identified a small or negligible change in item difficulty or discrimination for different option numbers [7,14-17], others have found decreases in difficulty and discrimination for smaller option numbers [3]. The results depend on the quality of the removed response options. From a theoretical perspective, the addition of distractor options which discriminate appropriately (negatively) should improve the overall item discrimination [18]. There may also be an increase in the difficulty of the item, because the additional option possibly increases cognitive load and the proportion of test-takers guessing the correct answer tends also to decrease with increasing option numbers.

Psychometric indices were not the only determinant of item quality. Educational value was also considered. Uniform response option policies (whether it is three or four or more) are at odds with the advice from Frary [19] and Swanson [20], who both argue that some questions invite particular sets of alternatives on curriculum grounds. By this rationale, educational or clinical related alternatives are included and irrelevant alternatives are not. For example, according to the RANZCOG Intrapartum Fetal Surveillance Guidelines [21] there are four broad categories of fetal heart rate deceleration. Any item assessing the ability of practitioners to distinguish the type of deceleration pictured in an accompanying cardiotocograph (CTG) might therefore have the four categories of deceleration as options. As cited earlier, there is no psychometric reason that all items must have the same number of options. In most content areas, it could be argued that there is no educational reason either. Tarrant, Ware and Mohammed provided the following synopsis.

So while in most circumstances, three options would be sufficient, item writers should write as many good distractors as is feasible given the content area being assessed. Additionally, when reviewing item performance on previous tests, test developers and item writers should not eliminate options that perform adequately simply to conform to a pre-set number of options [3].

This view, combined with the practice of including educationally important alternatives, might be termed an *item-level* approach to determining the number of response options.

For some items FSEP subject-matter experts reported difficulty in producing a plausible fourth option. It was reported to result in additional time being spent in item-writing workshops for what were typically minimal gains in psychometric quality. Further, the risk of introducing problematic options is arguably increased when subject-matter experts are required to add options which they would otherwise omit.

From the literature some basic conclusions emerge. Where the number of items is not fixed, maximising the number of items with three response options usually emerges as the best approach. This is conditional on gains such as time savings and improved content sampling. If the number of items is fixed or testing times are more invariant than the proportionality assumption predicts, the addition of plausible options can marginally improve the quality of the test, depending upon item content and option quality. Beyond psychometric considerations, the flexibility to add educationally or clinically important alternatives, in spite of low selection frequencies, provides an opportunity to bolster arguments in support of content validity.

We undertook the present study with the assumption that the number of questions will be held reasonably constant in a final version of the assessment system. While we did not have accurate information about response times, and acknowledge that such information would be useful, we did not see any potential reduction in the number of response options as an opportunity to increase the number of test items in the FSEP. Further, in the domain of fetal surveillance knowledge it has been reported that 25 to 50 questions might provide an adequate sampling of content [22]. On this basis, we believe that the FSEP 60 item test forms provide adequate scope for sampling critical content. The addition of extra items would have more influence on increasing reliability and decreasing measurement error, rather than addressing content shortcomings. As discussed in subsequent sections, decreasing measurement error remains an important objective in the FSEP context.

## Method

Three versions of a 60-item FSEP multiple-choice assessment were compiled. The first version contained four options per item, one correct option and three incorrect options. The second version contained three options per item, with one less incorrect option. This three-option version was constructed in the following way: for items that had been used in previous four-option assessment forms the least frequently chosen option was removed to construct a three-option item. If a four-option item contained a positively discriminating incorrect option with a reasonable selection frequency, then this was removed instead. These two criteria (less than 5% selection frequency and the sign of the distractor discrimination) appear to be the most commonly reported in studies concerned with identifying non-functioning distractors [3]. For a smaller number (11) of new four-option items that had yet to be trialled, subject-matter experts eliminated what they perceived to be the least plausible incorrect option, based on experience with similarly styled/structured items, to produce a three-option item. Cizek and O’Day [14] showed that subject-matter experts’ selections can be highly consistent with empirical data about these relative frequencies. In compiling the first two test forms, exactly the same items in terms of the item stem appeared in each test. The item order was also preserved across test forms to avoid order effects.

The third test was constructed as a mixed-option test form. This test was largely constructed from the items trialled in the fixed-option test forms. A total of ten three-option items were retained. A total of 38 items were sourced from the four-option test. These retained items represented the version of each item which discriminated best in the trial. Also, 12 new items were introduced. Of these, only three were completely new, whilst nine were items which underwent minor amendment. In order to avoid contamination by the new items only data from the subset of 48 common items are used to derive test and item indices.

Details about the test content and the target population are described in Zoanetti et al [23]. The three-option test was administered to 646 practitioners and the four-option test was administered to 763 practitioners. Test administration took place in a number of testing sessions. Fixed-option test booklets were distributed randomly across testing sessions so that an assumption of equivalent ability distributions could be supported.

The mixed-option test was administered to a different sample of 1044 practitioners from the FSEP target population. A comparison of values for various test statistics across the three 48 item subsets was then made.

A variety of indices of test and item quality were computed. These included: the mean difficulty of the tests in terms of classical test theory (CTT) item facility values, the mean of the item discrimination values, and the internal consistency index Cronbach’s [24] Alpha. Item indices included: CTT item discrimination (point biserial), item facility as the percentage of correct responses for an item, the number of non-functioning options, the item fit following scaling with the Rasch model, and the standard error of measurement (SEM) following scaling with the Rasch model. These latter two Rasch-based statistics were included given the intended scaling of FSEP assessment forms onto a common latent metric (refer to [23] for more details). Statistical tests were also conducted to analyse differences between a number of these indices including mean facility, discrimination and reliability across test forms. These tests included paired sample t-tests to evaluate facility and discrimination differences, and Feldt and Kim’s [25] test for comparing reliability coefficients from different test forms.

The Rasch measurement error for person scores is a function of the number of items and the targeting of each item’s difficulty to the estimated person ability. Unlike CTT, where a SEM is a property of the test and is assumed to be constant across all test takers, the SEM for person scores under the Rasch paradigm varies with a person’s scaled score (the estimate of a person’s latent ability). Our interest in this assessment context is that the SEM for a person should be minimized. More specifically, when a pass standard is established for the FSEP assessment, our interest will be in minimising SEM for practitioners scoring near pre-determined cut-scores. Small SEM values reduce the uncertainty surrounding decisions about whether test takers either exceed or do not meet specified standards. It also means that the measurement process will support decisions about a greater proportion of the test takers. Test takers for whom it cannot be determined with high likelihood whether they exceed the pass standard will require additional evidence to be considered about their competencies before any high-stakes decision can be made. For additional explanations of Rasch measurement error, we refer the interested reader to Schumacker and Smith [26].

## Results

The results for this study are presented in three sections. The first two sections detail differences in test and item statistics between the three-option and four-option test forms. The third section compares the statistical characteristics of the mixed option version against the fixed option versions of the test.

### Test statistics for the fixed-option forms

The first statistical comparison concerned the relative difficulty of items from the two test forms. In the present study, the mean CTT item facility on the three-option test was higher than the mean item facility on the four-option test by 5.7%. This difference was significant when calculated via a paired sample t-test of item facility across all items (t=-6.358, df=59, p<0.001). A total of 46 of the 60 items became easier upon removal of the least attractive incorrect option.

Also noted was a modest difference in the internal consistency index, Cronbach’s Alpha. The four-option test had an Alpha value of 0.791 while the three-option test had an Alpha value of 0.775. The difference in Cronbach Alpha values illustrates that the four-option test had slightly superior internal consistency. Feldt and Kim [25] developed a test for comparing reliability coefficients from similar tests. Their W statistic approximates to a central F-distribution with N_1_-1 and N_2_-1 degrees of freedom, where N_1_ and N_2_ are the two sample sizes, the critical F value was calculated as *F*_crit_ (645, 762) = 1.13 at *α* = 0.05. It was then determined that the test statistic *W* ≈ F = 1.08 < 1.13, indicating that the four-option form did not have a statistically significant higher reliability coefficient than the three option-form (p= 0.16).

Nonetheless, while there were no significant differences between the two fixed item forms, we determined how many additional three-option items of comparable quality might be needed to obtain an increase in Cronbach’s Alpha. Assuming equivalence of ability distributions of the practitioner samples, the implication of a larger Cronbach Alpha value is that the four-option test would provide more reproducible estimates of the relative ranking of practitioners. Importantly, for the three-option test to rival the four-option test in terms of this index, it is estimated by use of the Spearman-Brown [27,28] prophecy formula that an additional 6 items of equivalent quality to those already on hand would have been required.

The mean item discrimination of the two tests was also compared using a paired sample t-test. The mean of the differences was less than 0.01 and not significant (t=0.867, df=59, p=0.389). Interestingly, the non-parametric correlation of item discrimination values between the two forms was modest at 0.72. This suggested some re-ordering of the relative discriminating power of items had occurred following removal of the least functioning distractor. The standard deviation of the differences was 0.07, also suggesting the presence of reasonable variation at the item level. This highlighted the importance of examining changes across test forms at the level of individual items. The next section takes this approach.

### Item statistics for the fixed-option forms

The items were analysed and the following statistics were calculated: facility (percentage of correct respondents), item discrimination (r), response option (A, B, C or D) frequencies and missing response frequencies (percentage of respondents) and response option point biserial (Pt Bis) values. These values are displayed in Table 1.

Several things were evident from an inspection of the item analysis. Three items had not functioned well. These were item 1, item 9 and item 54. These were flagged for qualitative review and replacement in subsequent versions of the assessment. Net decreases in CTT item difficulty were observed for a total of 50 of the 60 items, supporting the theory that removal of negatively discriminating options will increase item facility. Only two items had increased in difficulty by what was considered a substantive, though arbitrary, amount (>5%) upon reduction to three options. These were item 26 (6.45% lower facility) and item 58 (14.70% lower facility). At the other extreme, the facility of item 37 increased 26.74% upon removal of the least functioning option. Net decreases in discrimination were observed for a total of 31 of the 60 items, representing a fairly even split. However, in some individual cases the reduction in discrimination was as large as 0.18 (item 53) and the increase as large as 0.16 (item 45).

**Figure 1:**
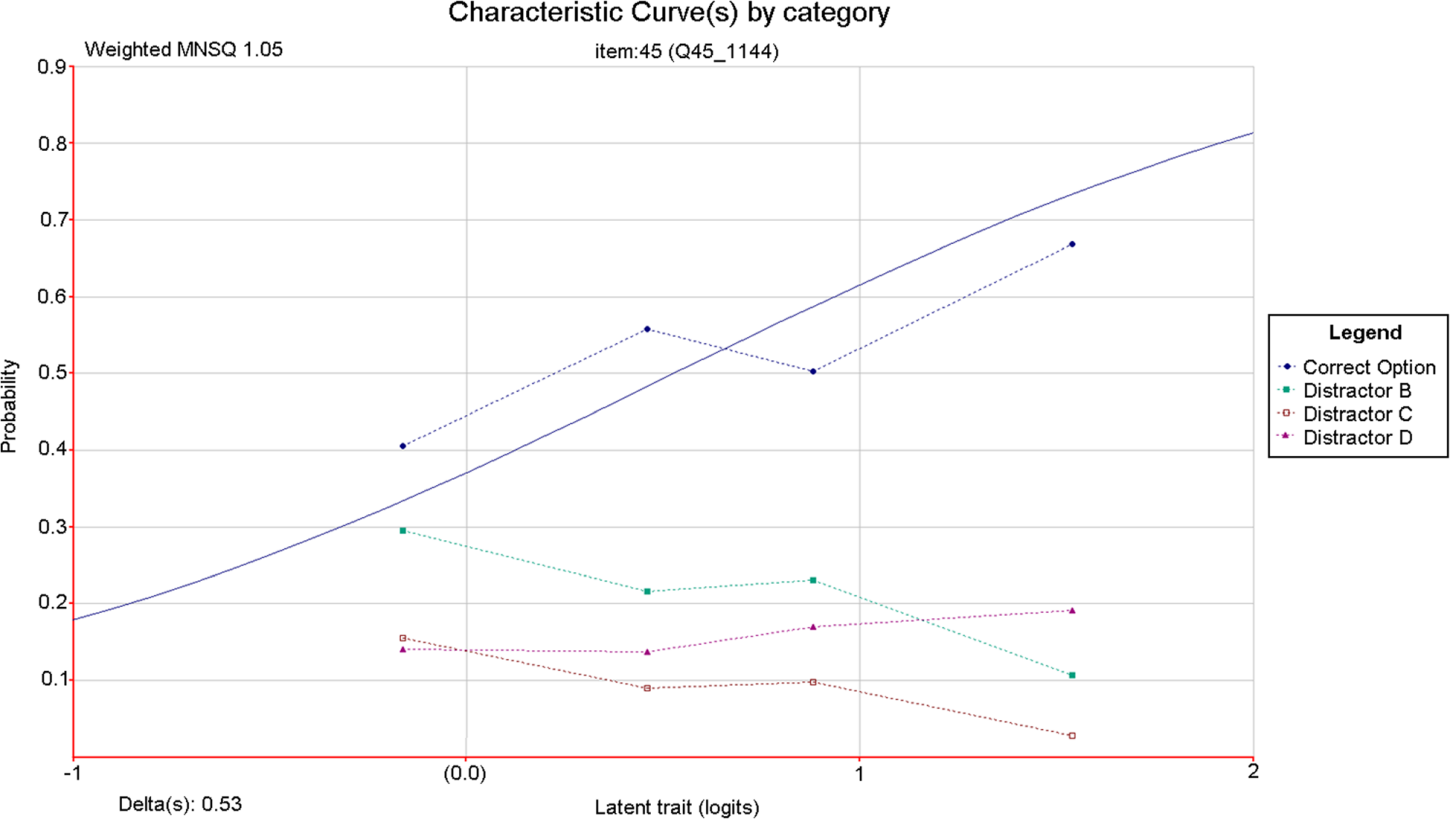
Relationship between candidate ability and probability of correct answer for a single test item with three distractors.

**Figure 2:**
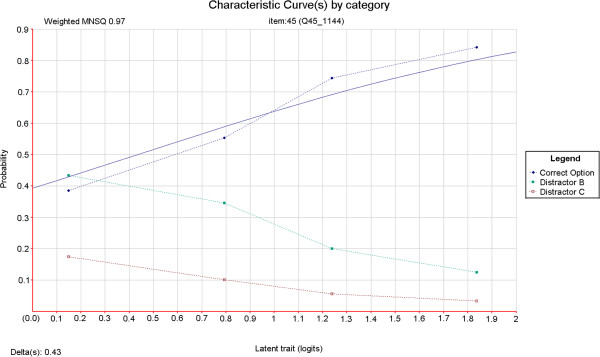
Relationship between candidate ability and probability of correct answer for a single test item with two distractors.

The average number of non-functioning options per item in the four-option test form was 1.18. The definition of non-functioning distracters used by Tarrant, Ware and Mohammed [3] was used in deriving these figures. This definition requires that an incorrect option is selected by less than 5% of test takers, or that an incorrect option discriminates positively. This latter criterion should be met with caution given that such statistics are sensitive to small numbers of respondents in low-response categories. Nonetheless these criteria are mirrored here. Item 45 is one example of an item for which the removal of the least effective distractor (this time on the basis of discrimination) resulted in a marked improvement in item quality. Item characteristic curves were produced for both versions of this item using ConQuest [29] item response modelling software (refer to Figure 1 and Figure 2). In a good item, the category curves for distracters should decrease in probability value with increasing test taker ability. In Figure 1 it can be seen that option D does not behave in this way. Its removal resulted in a sharp increase in item discrimination (refer to Table 1) and an improvement in the fit of the data to the item response model (refer to Figure 1 and Figure 2). An evaluation of this result and the specific item is outlined in the discussion section of this article.

### Comparing the mixed-option and fixed-option test forms

In the following comparisons, statistics were derived for a subset of 48 out of the 60 items. These items remained unchanged in terms of content and ordering across test forms.

The results in Table 2 suggest that, in this case, the mixed-option format was superior in terms of reliability and mean discrimination. Interestingly, it was found to be easier than the two fixed-option tests. Feldt and Kim’s [25] test for determining whether reliability coefficients from independent tests are equal was again applied to test the alternative hypothesis that the reliability of the mixed-option test form was greater than that of the four-option test form. The critical F value for this test was calculated as *F*_crit_(762, 1043) = 1.12 at *α* = 0.05. It was then determined that the test statistic *W* ≈ F = 1.17 > 1.12, illustrating that the difference is significant at the 5% level (p-value = 0.02).

**Figure 3:**
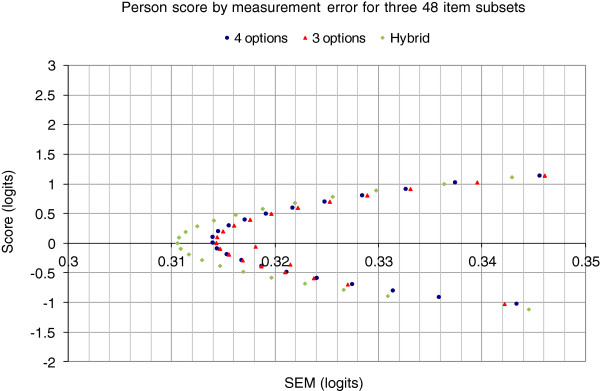
Plot of Rasch person score versus associated Rasch measurement error.

Next we examined whether there are differences in the measurement error surrounding estimated person scores. The mixed-option test form reduces measurement error, albeit slightly (Figure 3). In this context, given the consequence of the test score interpretation, even small reductions are important but it is likely to be more efficient to increase the number of items in the instrument.

## Discussion

It appears that there are advantages to be had in using a mixed option number mode of testing. At least this appears to be the case when comparing three-option and four-option alternatives. At the item level it is possible to increase the discrimination and content validity of individual items by adding plausible options and avoiding problematic options. This provides a basis for an evidence-based approach to item development where the number of response options for each question is determined independently. The approach can be applied at several important junctions during the assessment design and analysis process. First, item writers can apply the policy during item construction. Second, subject-matter experts can apply the policy during item panelling. Third, the policy can also be referred to during item analysis review. The following discussion examines how this policy might be implemented in the FSEP context and more broadly. It is evident that a rigid adherence to a fixed number of options regardless of the quality of options is counter-productive in terms of the quality of the psychometric information to be obtained from test administration.

Instructions to item writers might explain that only options which are educationally important and plausible should be written until a minimum of three are produced. If more are immediately forthcoming they may also be added. The minimum of three is chosen based on studies revealing that reliability increases tend to be more pronounced between two-option and three-option tests than between three-option and four- or five-option tests [18]. Preventing item writers from labouring over a fourth or fifth option is one way to extract the benefits of time savings afforded by using fewer options.

The FSEP item writing process is presently conducted using a round table audit of each newly constructed item. During this process options which are implausible are challenged and replacements are suggested. The item-level policy promoted in this article would result in a subtle change to the present process: If the task of replacing a challenged option for a four-option item became unfeasible, it could be abandoned and the item accepted as a three-option item. Alternatively, additional plausible options could be put forward by panel members at this time. The process would result in items with three or more response options.

Finally, item analysis data could be used to affirm that response options are at the very least discriminating appropriately. For low frequency options, caution with regard to sample sizes will be needed. Small, positive biserial values may emerge by chance if the numbers of test takers selecting certain response options is small. Recommendations that options selected less frequently than 5% should be removed from items should be made conditional on item facility. In some examination contexts, where mastery of particular knowledge or skills is expected and therefore included in the corresponding assessment blueprint, there may be a reasonable number of items with high facility. In these cases it is recommended that options not be discarded on the basis of frequency data alone. As reported in the results section, 16 items (26.7%) had zero non-functioning distracters, 22 items (36.7%) had one, 17 items (28.3%) had two, and five items (8.3%) had three. These results are not considered meaningful without first examining the facility of the items from which they arose. For example, the minimum facility of the five items with three non-functioning distractors is 87.68%. It is completely reasonable that items with very high facility cannot support many functioning distractors [6]. Yet these items are still necessary for fulfilling the content coverage requirements of the assessment blueprint. Another implication of these results is that for approximately three quarters of the four-option items, at least one non-functioning distractor was available for exclusion.

As identified in the results section and depicted in Figure 1 and Figure 2, the discrimination and model fit of item 45 improved markedly upon removal of a non-functioning distractor. Fit to the Rasch model is usually determined in two ways. The first is called infit and is the value of the mean squared deviation from the expected response pattern weighted by the item variance. The second is called outfit and it is determined by the unweighted mean squared deviation from the expected response pattern. The unweighted fit statistic is more sensitive to outliers within the data. The lower infit (weighted mean square) value indicates that the responses to the item have become less random and instead are better aligned with test taker ability as predicted by the measurement model. In this case, the three-option version of the item is of acceptable quality and need not be further modified. Qualitative review of this item revealed that option D (“This CTG is not reflective of the fetal condition”) could be considered an ‘easy out’ option. As discussed, this option appealed to a reasonable number of test-takers irrespective of ability. Removal of this option effectively forced test-takers to choose from options which better revealed their level of understanding of the fetal physiology indicated by the CTG. Item 32 similarly contained this ‘easy out’ option as its option D, and also exhibited improved discrimination upon its removal (Table 1). This information has since been fed back into item writing workshops, where inclusion of ‘opt out’ options to make up option numbers has been discouraged. Generally, the results presented in this article are consistent with those reported by Cizek, Robinson and O’Day [14] where test-level variation is modest but item-level variation can be considerable, following a reduction of one response option.

Given that items would routinely be stored in an item bank, and potentially rotated in and out of test forms, their optimisation is an important component of the FSEP. This is an important point which suggests that test or aggregated statistics like mean facility or discrimination should not form the basis of item writing or test construction policies alone. Cizek *et al*. [14] have made similar remarks.

This study has provided some useful empirical information from which to determine a policy for FSEP test item writing. That stated, a number of assumptions are made and a number of limitations have been identified. These are outlined in the following paragraphs.

One assumption made throughout the study is that the samples of practitioners taking the different assessment forms are representative of each other and of the FSEP target population. The large sample sizes and the relatively random distribution of three- and four-option booklets to different testing groups would go some way to ensuring this. Nonetheless, this was recognised as a source of error when making comparisons between item and test performance indices across test forms. This would also lead to some uncertainty concerning the generalisability of these results.

The second part of this study comparing the fixed-option and mixed-option forms has the design limitation that a subset of 12 questions was not common across test forms. Despite restricting the analysis to the common subset and majority, any influence of the smaller disparate subset on test taker responses to the common questions cannot be accounted for. Based on efforts during test construction to avoid inter-item dependencies, it is hoped that any influence would be small.

A further limitation of this study is that the approach taken is post-hoc. That is, options were removed that were non-functioning. This is not the same as recommending writing just three options, since there is likelihood that item writers will not purposefully include non-functioning options. In other words, during item writing, it is often unknown which option will be non-functioning. Therefore the impact of writing only three options could be more severe if items were constructed that way. This is another limitation of the study design, in that it produced three-option items which, by design, should be of a higher quality than those constructed by item writers aware that three-option items are sufficient.

The interplay between option number, test length and test reliability deserves further attention. The theoretically predicted result that approximately 6 additional three-option items might be needed to compensate for the reliability reduction from four-option items is rather inconclusive. Whether test takers could reasonably answer 66 three-option questions in the time it would take to answer 60 four-option questions is an empirical question for this context. Based on a meta-analysis by Aamodt and McShane [30] it could go either way. They estimated that in the time it would take to complete a test with 100 four-option questions an additional 12 three-option questions could be completed (so 112 three-option items in total). Another estimate reported in Rogausch, Hofer and Krebs [6] suggests that about three or four extra items per hour of testing time could be answered based on removal of one option per item. The FSEP test duration is one hour, suggesting that an extra 6 three-option items may not necessarily be accommodated in the testing period. A follow up study looking at the time taken to complete the various test forms would provide useful additional information in this context. Following this, formulae for projecting the increase in reliability owing to increased item numbers could be used to model how mixed-option test forms with different proportions of three-option and four-option items might perform.

Finally, the empirical components of this study apply to a particular sample of items from a broader item bank. It is not known to what extent the results would generalise to other contexts within medical education and beyond.

## Conclusions

In this study we sought to determine a policy for item development and test construction for the FSEP assessment. A review of literature and existing assessment practices identified a number of feasible approaches, each underpinned by various traditions, assumptions and empirical findings. The commonly reported finding that test difficulty decreases slightly and mean item discrimination remains unchanged when the least functional distractor is removed from all items was supported in the FSEP context. The finding that items perform no worse with three options than with four options when the least functional distractor is removed was not supported in the FSEP context. Instead, there was appreciable variation at the individual item level. These findings mirror those in another medical education assessment context [14], and contribute to the idea that these trends are generalisable. This discouraged the recommendation of a blanket policy for the number of options. The view was taken that where plausible and educationally important options could be included in an item they should be, without regard for the total option number. Indeed, for other items, where specifying more than two plausible options would be difficult, item writers would not be obliged to spend excessive time trying to construct additional, potentially poor quality options. These policies were seen as the most evidence-based approach for maximising the quality of the FSEP test.

## Competing interests

The authors declare that they have no competing interests.

## Authors’ contributions

Nathan Zoanetti and Patrick Griffin conducted and interpreted the statistical analyses of items and test forms. Mark Beaves coordinated the associated test development and validation program. This included assembling expert practitioner groups, and compiling and administering test forms. Both Mark Beaves and Euan Wallace led the item writing workshops and led discussions about critical practitioner competencies and educational outcomes in light of the relevant guidelines. All authors participated in the writing and revision of the manuscript. All authors read and approved the final manuscript.

## Pre-publication history

The pre-publication history for this paper can be accessed here:

http://www.biomedcentral.com/1472-6920/13/35/prepub
